# NORMALIZING COGNITIVE EVALUATIONS IN ADULTS: GETTING PEOPLE IN THE SCREENING PIPELINE EARLY

**DOI:** 10.1093/geroni/igac059.1871

**Published:** 2022-12-20

**Authors:** Laura Mehegan, Chuck Raineville

**Affiliations:** AARP, Washington, District of Columbia, United States; AARP, Washington, District of Columbia, United States

## Abstract

To understand how adults and healthcare providers perceive a dementia diagnosis and cognitive evaluations, AARP fielded a nationally representative survey of adults aged 40 and older (N=3,022) along with a companion survey of healthcare providers in a position to diagnose dementia (N=500). Like healthcare providers, Americans aged 40 and older understand the benefits of diagnosing dementia at an early stage, 81% agree that early detection would motivate them to engage in healthier behaviors to slow the progression of the disease. Adults look to healthcare providers as authorities to determine when a cognitive evaluation should be triggered with 63% saying they would get an evaluation if their doctor recommended it and 49% of healthcare providers saying evaluations should be done if the doctor feels it is necessary and 39% saying it should be done yearly beginning at age 65. The research also showed that most adults (76%) want to know if they have dementia. Additionally, majorities of adults would engage in healthy behaviors if they knew it’s good for their brain health. Given the desire to prevent and improve outcomes, routine screening for cognitive function should be as commonplace as getting a screening colonoscopy. Unlike a colonoscopy, routine screening for cognitive function is not a normalized process yet. Data from both populations suggest an opportunity for routine evaluation due to a shared understanding of the long and short-term benefits of screening.

